# Self-Assembly Magnetic Micro- and Nanospheres and the Effect of Applied Magnetic Fields

**DOI:** 10.3390/nano11041030

**Published:** 2021-04-17

**Authors:** Angelos Mourkas, Angeliki Zarlaha, Nikolaos Kourkoumelis, Ioannis Panagiotopoulos

**Affiliations:** 1 Department of Materials Science and Engineering, University of Ioannina, 45110 Ioannina, Greece; kikizar@yahoo.gr (A.Z.); ipanagio@uoi.gr (I.P.); 2 Medical Physics Laboratory, Medical School, University of Ioannina, 45110 Ioannina, Greece; nkourkou@uoi.gr

**Keywords:** magnetism, self-assembly, nanospheres

## Abstract

The impact of in-plane and perpendicular magnetic fields on the spatial arrangement of superparamagnetic nanospheres is explored. We utilize nanosphere self-organization methods like Spin Coating and Drop-Casting in the presence of magnetic fields. In this way, the additional parameter of the long range magnetic dipolar interactions is introduced to the competing nanosphere–surface and nanosphere–nanosphere interactions, which control order and agglomeration. We present a comparative analysis of the self-assembly characteristics with respect to the different methods and the effect of the applied field in different directions. Under zero field perfect hexagonal arrays can be obtained by spin coating. Parallel applied fields tend to create directional patterns, while perpendicular favor 3D-accumulation.

## 1. Introduction

Modern magnetic-material applications require homogeneous microstructures consisting of monodisperse magnetic entities. A facile route towards such microstructures is to exploit other materials as templates of model nanostructures via a “bottom-up”, low-cost approach. Monodisperse nanosphere single-layers that tend to form 2D-hexagonal close packed patterns can be utilized either as templates for the deposition of “nanocaps” or as masks for the formation of triangular-like islands [1,2,3,4].

The underlying mechanisms of spontaneous nanosphere organization involve the formation of small aggregates of nanoparticles into larger structures, under the influence of capillary particle–particle, particle–substrate, and hydrodynamic forces [2,3,4,5,6,7]. Recently, Chandramohan et al. [8] developed a spin-coating method for the successful self-assembly of NPs with respect to hexagonal packing density and large area surface coverage. Crucial microscopic parameters are the centrifugal force, gravity, surface tension, and evaporation rate of the fluid and the substrate’s hydrophilicity. These are controlled by the various solvent and nanosphere concentrations in the solution, the rotation speeds, and use of appropriate surfactants. They achieved high, defect-free, area coverage using variable rotation speed, under constant acceleration, allowing for optimal performance during the different stages of spin coating. A similar methodology had been proposed previously and studied both experimentally and theoretically [9].

A simpler approach for balancing between gravitational forces and suspension’s evaporation rate, is based on drop casting [1,10,11]. By “cleaning” and tilting the substrate almost vertically, hexagonal packing with satisfactory area coverage can be achieved. The optimization is based on pinning the NPs to the substrate during fluid movement. Fluid evaporation can be delayed by drying in ambient conditions. Independently of the technique (spin coating, drop casting, and dip coating), fast evaporation rate of the solution may negatively affect the extended structural coherence and thus the quality of the monolayers [3,12,13].

The use of magnetic forces during these processes has been less studied. Dipolar interactions are known [14,15] to lead to the formation of one-dimensional chains and rings and may not seem promising for formation of regular arrays. However, magnetic forces can be used to obtain novel and interesting types of 2D and 3D organization [16,17].

Jadav et al. have used magnetic nanofluid to induce bi-dispersed suspension of non-magnetic particles into colloidal chain, triangle, rectangle, and ring-flower configurations [18]. Abelmann et al. have demonstrated that 3D structures can self-assemble by taking advantage of dipolar forces [19]. Ahniyaz et al. investigated how the long-range order of maghemite nanocube superlattices can be controlled using a modulated magnetic field [20]. All these open the possibilities to prepare tailor-made magnetic metamaterials [21,22].

Here, we present the utilization of the above self-organization methods, for the effective self-assembly of superparamagnetic polystyrene nanospheres, with diameter range of 0.25–1.43 μm. Due to the significant aggregation of magnetite NPs, a polystyrene matrix is suggested for the NPs to be self-assembled [23,24]. We report on the formation of monolayers with respect to 2D hexagonal packing, monolayer formation, and substrate coverage. In addition, a comparison of the same organization methods is given with and without in-plane and perpendicular applied field.

## 2. Materials and Methods

Aqueous solutions of polystyrene micro/nanospheres of diameters 0.27–1.31 μm, with incorporated iron oxide superparamagnetic nanospheres (MSNs) were obtained from MicroParticles GMBH. Iron oxide particles that are commonly employed for biomedical and similar applications include both magnetite (Fe_3_O_4_) and maghemite (γ -Fe_2_O_3_). The magnemite is derived from magnetite by adding vacancies (one-third of an atom out of the 2 atoms of the Fe^+3^ sites). These two phases are practically indistinguishable by most characterization techniques (with the exception of Mossbauer spectroscopy), especially in such small particle sizes, and in-between phases can exist. The exact crystal phase, is not crucial for their functionality, as it regards the aims of the present study. It is their superparamagnetic nature that permits them to be driven by external fields, while at zero field, they become essentially non-magnetic. Thus they will not agglomerate uncontrollably but only to the degree that the strength of the external field imposes.

**Figure 1:**
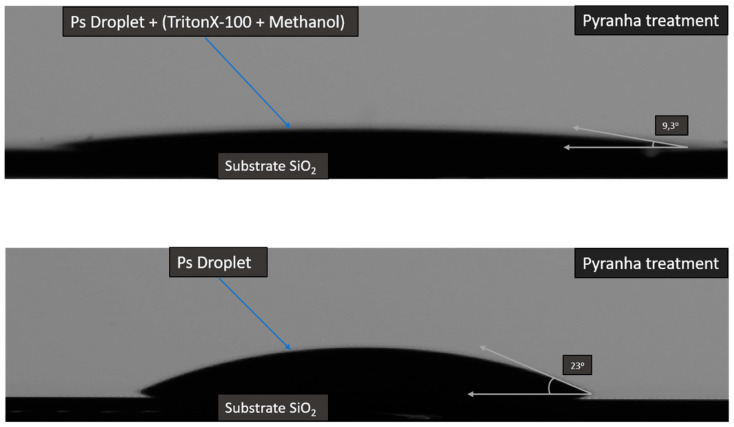
Contact angle measurements showing the improvement of wetting upon Triton X-100 surfactant addition in the aqueous suspension.

The MSNs were deposited, as 1 × 1 cm^2^ pieces of thermally oxidized Si wafers. These were covered by a 500 nm oxide layer, which is functionalized to improve wettability. The substrates were treated by a “piranha” solution (H_2_O_2_ and H_2_SO_4_ at a ratio of 1:1) to increase the surface’s hydrophilicity. After that, the substrates were washed with ultrapure water and sonicated in an isopropyl alcohol bath for 10 min followed by drying with N_2_ to remove the residual acid. This surface treatment was crucial in order to obtain a homogeneous solution deposition on the substrate’s surface. The hydrophilicity was quantitatively evaluated by contact angle measurements. On the untreated SiO_2_ surfaces, the solution had a contact angle of 46°, suggesting a mild wettability. After the piranha treatment, the contact angle was lowered to 23°. The contact angle could be further lowered down to 10° by utilizing surfactants as Triton X-100 and sodium dodecyl sulfate (SDS). Triton X-100 was diluted with methanol at a ratio of 1:400 and then mixed with the MSNs solution. This straightforward procedure resulted in a high boiling point solution due to the presence of the Triton X-100. SDS, on the other hand, was utilized both before and after the MSNs solution was deposited on the surface with the drop casting method. The substrates were left in a 10% aqueous SDS suspension for 24 h. Typical contact angle measurements are presented in Figure 1.

The droplet spreads on the surface in a more efficient way when using Triton X-100. Therefore, for the spin coating experiments, we suggest mixing of the initial aqueous solution with a surfactant; SDS is better suited for dip coating experiments similar to Rybczynski et al. [25] who employed a method of self-assembly based on a liquid-gas interface with SDS dipping.

Two different methods were utilized for the solution application to the substrate’s surface and for the MSNs self-assembly: drop-casting and spin-coating.

**Figure 2:**
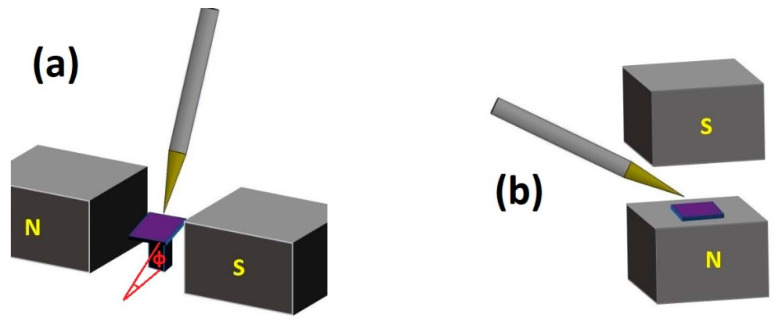
Schematics of drop casting in a magnetic field (**a**) under an applied field of 0.11 T parallel to the substrate, which is tilted by an angle of φ to the horizontal plane (**b**) under a field of perpendicular to the substrate.

Drop casting was performed using either the aqueous nanoparticle suspension as is or mixed with methanol and Triton X-100. Piranha treated substrates were placed on top of a base with variable inclination, ranging from 10° to 40°. To achieve magnetic organization, a small electromagnet was used to generate a magnetic field parallel to the substrate and perpendicular to the direction of the inclination. A schematic of the experimental setup geometry is depicted in Figure 2.

**Figure 3:**
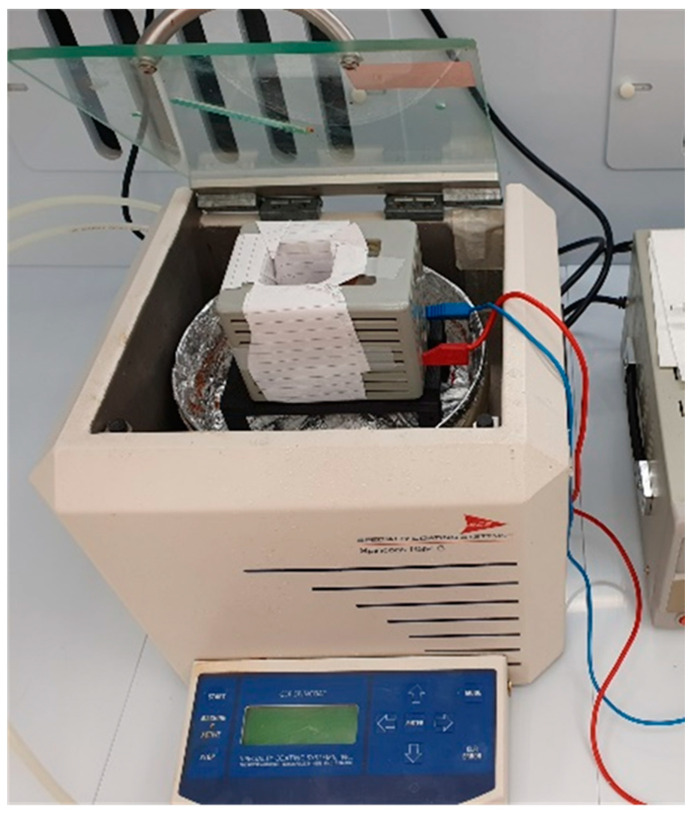
Spin coating configuration for sample rotation under perpendicular magnetic field. We utilize a current source and a coil, which is placed inside the spin coating with a special 3-dimensional—printed base, replacing the Spin Coaters’s sample holder.

In spin coating experiments, under perpendicular field, the surface-treated substrate was rotated on the platform of the spin coater, which was placed in the middle of a coil. For in-plane field application, the sample was rotated until the solution expands on the surface; then, it was removed from the spin coater and left to settle under the field on horizontal platform inside a coil. For the Triton X-100-treated samples, rotation speeds up to 8000 rpm with a standard acceleration rate of 200 rpm/s were used. The experimental setup is displayed in Figure 3.

For the observation of the surface morphologies of the samples at different scales, we used a Zeiss Axio Imager A1m optical microscope fitted with a Canon 80DDSLR and a Scanning Electron Microscope (SEM, JEOL Ltd., Tokyo, Japan). The magnetic properties of the MSNs were studied using a vibrating sample magnetometer (VSM) of Lakeshore Cryotronics Inc. (Westerville, OH, USA). The morphology in small scale was studied with an atomic force microscopy (AFM) in tapping mode with a Bruker Multimode 3D Nanoscope (Ted Pella Inc., Redding, CA, USA) using a microfabricated silicon cantilever type TAP-300G, with a tip radius of <10 nm and a force constant of approximately 20–75 N m^−1^.

## 3. Results and Discussion

### 3.1. Magnetic Measurements

**Figure 4:**
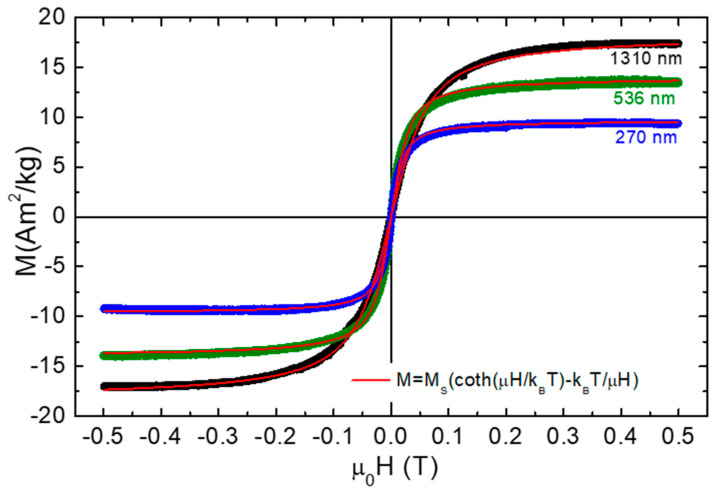
Magnetization curves for polystyrene/iron oxide spheres of different diameters, measured at room temperatures. The red lines are fits to the Langevin function. Ms is the saturation magnetization, k_B_T is the thermal energy taken at T = 290 K, and μ is the total magnetic moment of each particle.

In Figure 4, the magnetization curves obtained at room temperature with the help of a VSM are shown. There is no hysteresis, and the curves can be fitted to the Langevin function. This proves their superparamagnetic nature. Superparamagnetism is important for their functionality, in the sense that in a moderate field (e.g., 0.1 Tesla), they are already magnetized. On the other hand, at zero field they become essentially non-magnetic and would not tend agglomerate uncontrollably. The Langevin function fitting gives a magnetic moment (μ) ranging from μ = 16,600 μ_Β_ for the larger polystyrene spheres to 51,100 μ_Β_ for the smaller ones. This means that the larger polystyrene particles incorporate smaller iron oxide nanoparticles. Thus, the drop of saturation magnetization of the composite polystyrene/iron oxide spheres at smaller sizes must be assigned to reduced iron oxide content. The saturation magnetization of magnetite nanoparticles depends on the preparation conditions [26,27]. Some estimations can be done assuming a typical saturation magnetization value of 78 Am^2^/kg and the bulk density which gives M_S_ = 405 kA/m. Then, the weight percent of the magnetic phase is estimated to 12.5%, 18.2%, and 23.8% for the 270 nm, 536 nm, and 1.31 μm spheres, respectively. For spherical magnetic nanoparticles, the diameter is given by, $D=\sqrt[{3}]{\frac{6}{\pi }\frac{\mu }{M_{S}}}$ which yields 13.1, 11.7, and 9 nm respectively. 

### 3.2. Drop-Casting

#### 3.2.1. Self-Assembly under Zero Magnetic Field

**Figure 5:**
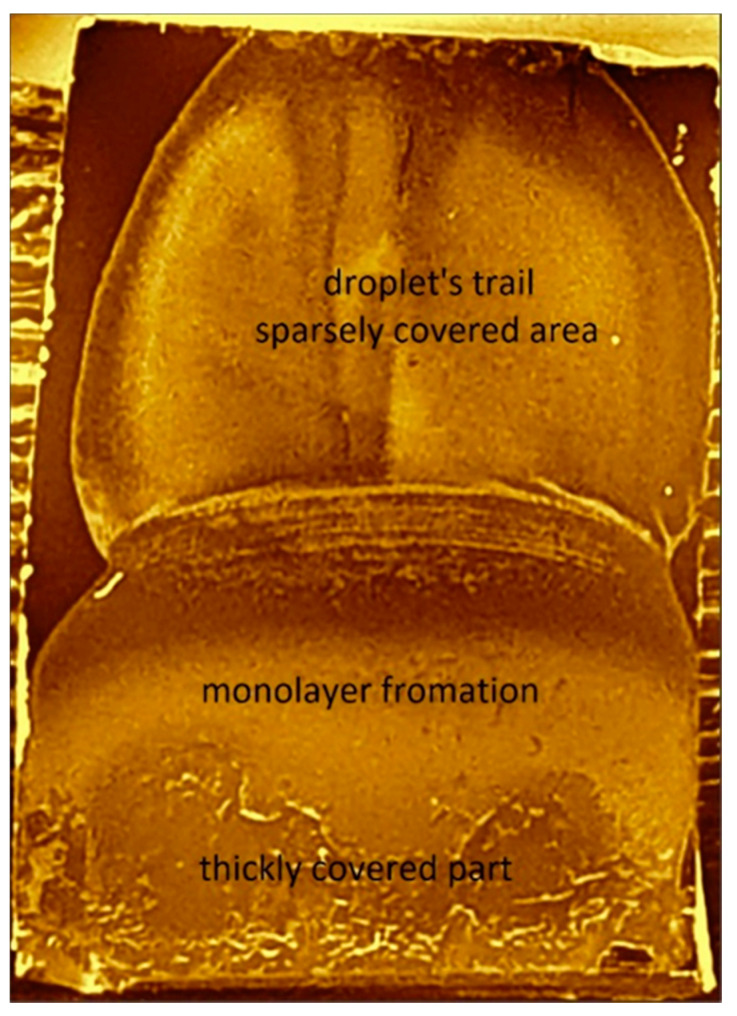
Substrate regions of different coverage formed upon drop casting at an angle.

**Figure 6:**
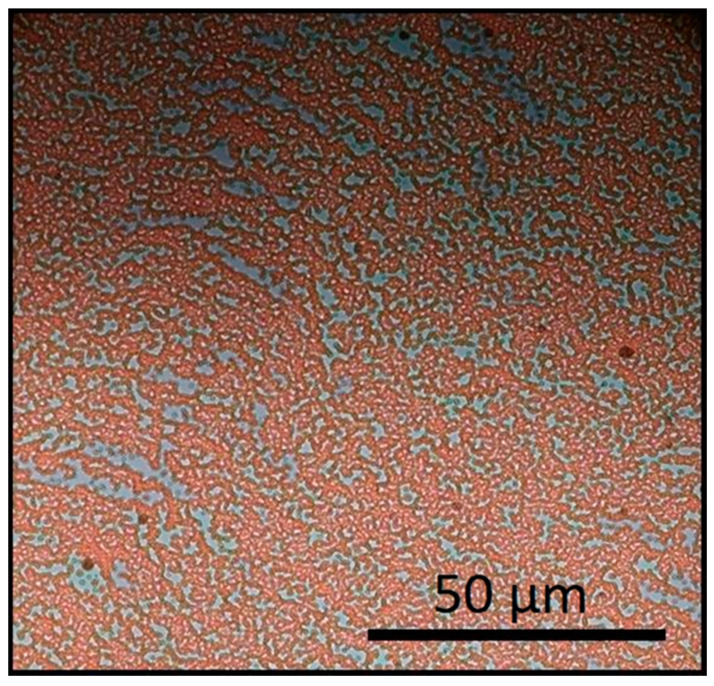
Self-assembly patterns of 536 nm diameter iron oxide superparamagnetic nanospheres (MSNs) under zero field.

To achieve small substrate sizes, the typical approach is drop casting of the aqueous nanoparticle suspension (without a surfactant addition) on the substrate’s surface which is placed at an angle to the horizontal plane. The solvent drying rate and wetting behavior determines the kinetics of this process. The inclination of the substrate surface allows the optimization of the velocity of the droplet sliding motion and its asymmetric deformation in combination with the evaporation rate to achieve the largest possible monolayer covered area. Too high inclination will lead to accumulation of the material at the bottom edge of the material, while on the other hand, too low inclination results in thick multilayer MSNs stacks. Monolayer formation is based on the residual material along the trail of the sliding droplet, and it is limited within an intermediate area (≈3 mm) between the sparsely covered upper part and thick material accumulated at the lower part (Figure 5). Then, structural coherence of the hexagonal arrays spans over 20–30 nanospheres and coexists with uncovered areas (Figure 6).

#### 3.2.2. Self-Assembly in the Presence of Magnetic Field

The same drop-casting process was also performed under external magnetic field applied along two different directions: in–plane and perpendicular to the sample’s surface. The applied field’s strength was 0.11 Τ unless otherwise noted. Taking advantage of the superparamagnetic nature of the MSNs, different kinds of organization are achieved as follows.

- In-Plane Field

**Figure 7:**
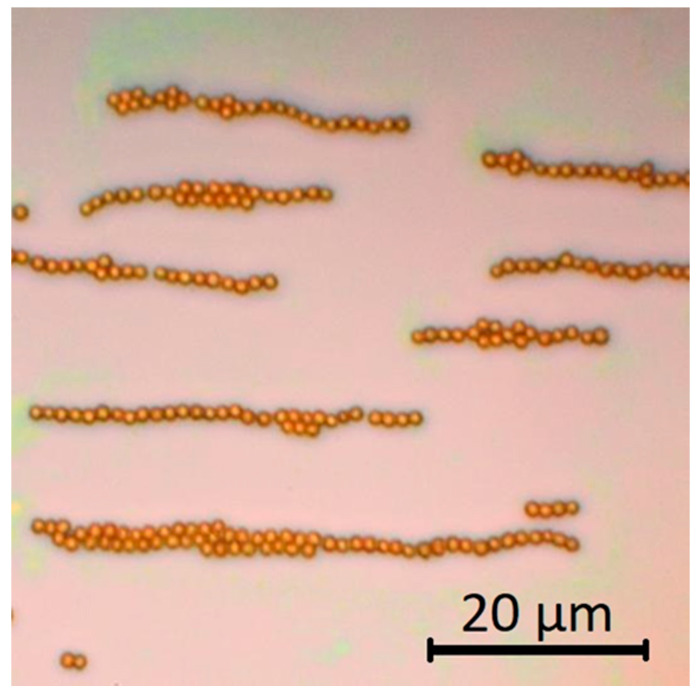
Line formation of 1.31 μm diameter MSNs under in-plane applied field allocated sparsely on the substrate. The applied field is 0.11 T along the horizontal direction.

As opposite magnetic poles are created on the opposite ends of each sphere, along the field direction, the tendency of magnetic nanoparticles to form lines can be explained from these dipolar interactions or equivalently as a result of magnetic flux closure. This kind of structures are observed in sparsely covered areas (Figure 7).

**Figure 8:**
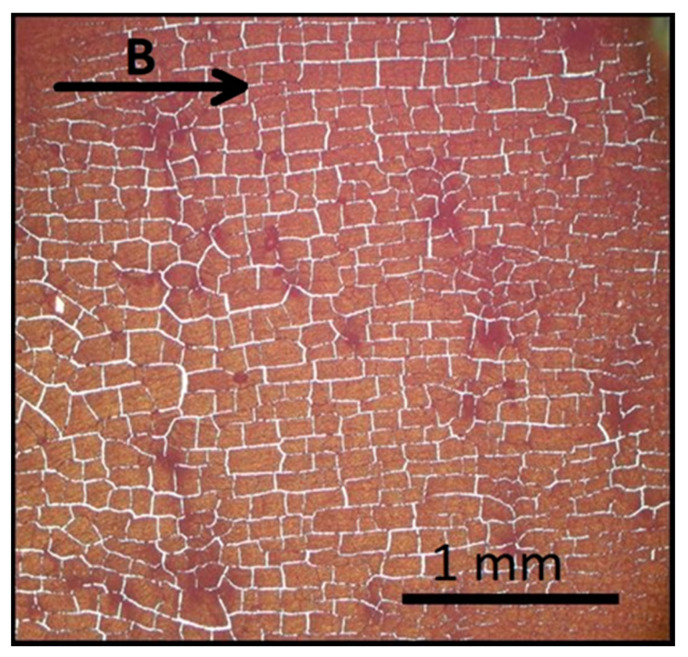
Formation of orthogonal blocks of 536 nm diameter MSNs arrays under an in-plane applied field deposited at φ = 10°. These blocks appear in densely covered areas of the substrate. The direction of applied field is indicated by the arrow.

This is a common behavior of MSNs when they assemble in a solid–liquid interface [17]. At lower deposition angles, like φ = 10° (Figure 8), MSNs of 536 nm diameter form large (in μm) rectangular blocks organized along the applied magnetic field direction. Their structural coherence spans with lengths of 0.2–0.3 μm. The “cracks” between the blocks are attributed to the evaporation of the water solvent.

- Perpendicular Field

**Figure 9:**
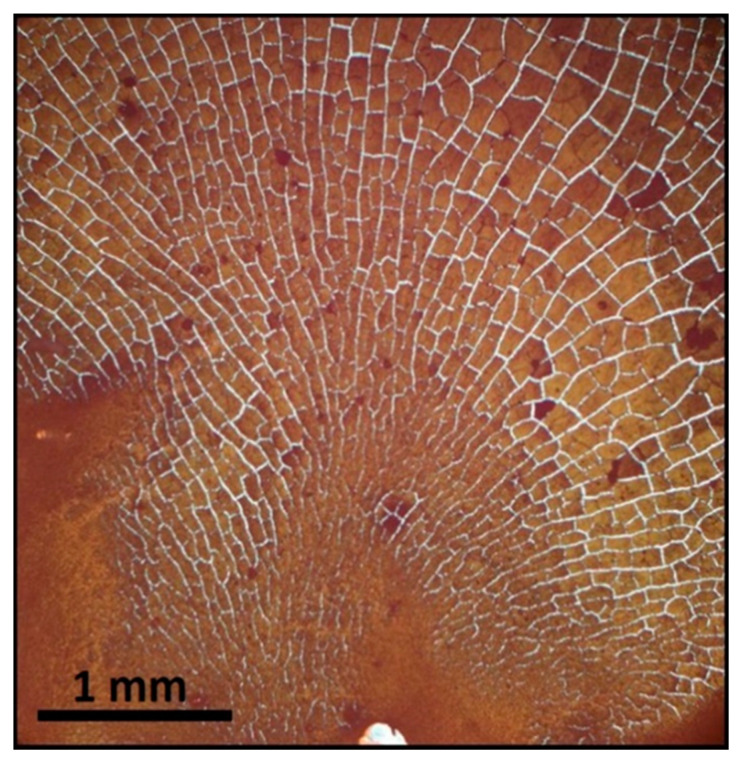
Formation of orthogonal blocks by 536 nm diameter MSNs arrays under a perpendicular to the substrate applied field B = 0.06 T.

The application of a magnetic field perpendicular to the substrate’s surface, results to the appearance of pyramidal 3D structures. The field was applied simultaneously with the solution drop casting on the surface. Different values of magnetic field, H_1_ = 0.06 T, H_2_ = 0.1 T, and H_3_ = 0.5 Τ were applied. This process results in increased concentration of the MSNs at the center of the sample. Here, also blocks of particles are formed as shown in Figure 9, for MSNs with 536 nm diameter but with irregular shapes when compared to those obtained by in-plane field. Since the field is out of plane, these flakes do not exhibit strong directionality within the substrate plane and tend to form radial patterns. Using stronger fields, nanosphere accumulation is enhanced at the center, and some of the blocks flake-off the substrate due to the strong attraction of the field.

### 3.3. Spin Coating

**Figure 10:**
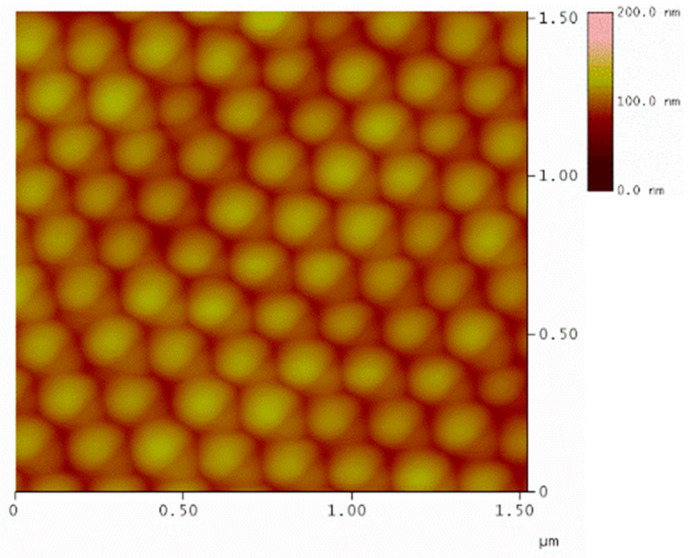
Surface topography obtained by atomic force microscopy (AFM) microscopy with height analysis. The Polystyrene nanosphere diameter is in excellent agreement with the nominal.

According to the process described by Chandramohan et al. [8], we have prepared samples of different diameter. The rotation speed extended from 150 to 8000 rpm. The SC routine is summarized in Table 1. The effectiveness of the process was checked on non-magnetic PS nanospheres with 170 nm diameter and yielded high-quality hexagonal arrays, as presented in Figure 10.

We have applied this method using different MSNs solution and surfactant ratios. Prior to the all the experiments, the substrates were cleaned and dried according to the procedure described in Materials and Methods section. The self-organized spheres formed a dense 2D-hexagonal grid in all cases.

#### 3.3.1. Spin Coating under Zero Field

**Figure 11:**
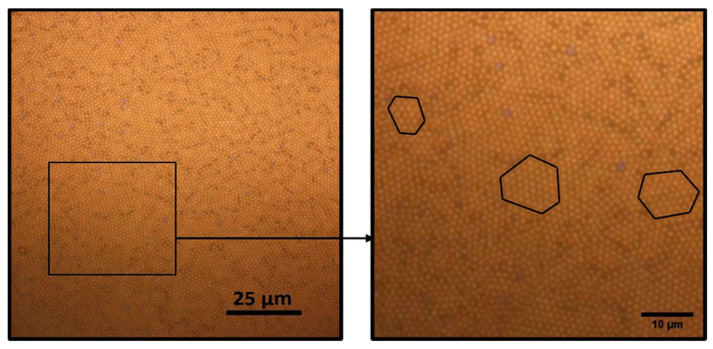
Self-assembly of spin coated 1.31 μm diameter MSNs to hexagonal monolayer arrays.

For this process, the optimal ratio of MSNs solution ratio to the surfactant solution (Triton X-100 + methanol) was found to be 4:1. The structural coherence of the hexagonal lattice is of the order of 10–30 spheres. Random voids among the MSNs configurations persist but in the form of point defects and not as extended areas as it was evident with the drop-casting technique (Figure 11). By scaling down to 10 μm, one can more efficiently observe the formation of these small hexagonal areas. Similar behavior is presented in Colson et al. [9] regarding the formation of small or large monolayer HCP ordered areas. In our case, we observed small hexagonal lattices with defects in between them, which restricts the structural coherence in the order of a small number of spheres. The defects are mainly dislocations, and they prevent a continuous large-scale formation of close-packed hexagonal lattice of MSNs. These small hexagonal lattices are attributed to the fast evaporation of the solvent [28].

#### 3.3.2. Spin Coating in Magnetic Field

- In–Plane Field

**Figure 12:**
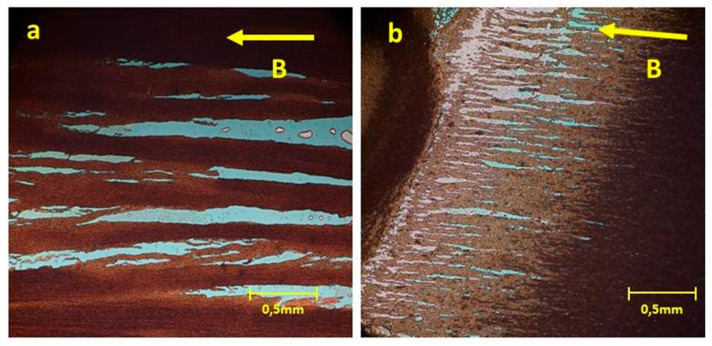
MSNs configurations obtained by drying ex-situ under the application of an in-plane magnetic field following spin-coating deposition of their aqueous solutions: (**a**) 536 nm diameter and (**b**) 1.31 μm diameter.

By applying the field in-plane to the sample’s surface, the MSNs are expected to align along the field’s direction. Following the same routine as before, adjustments were needed to achieve the MSNs self-assembly. The first stages of the SC are required for the solution thinning. The SC processes are terminated at stage 3 (Table 1) before the MSNs settle to their final positions. The deposited solution is left to dry under a magnetic field of H = 0.11 T. In Figure 12a, MSNs with 536 nm diameter are assembled in arrays of thick lines, along the direction of the external field.

- Perpendicular Field

**Figure 13:**
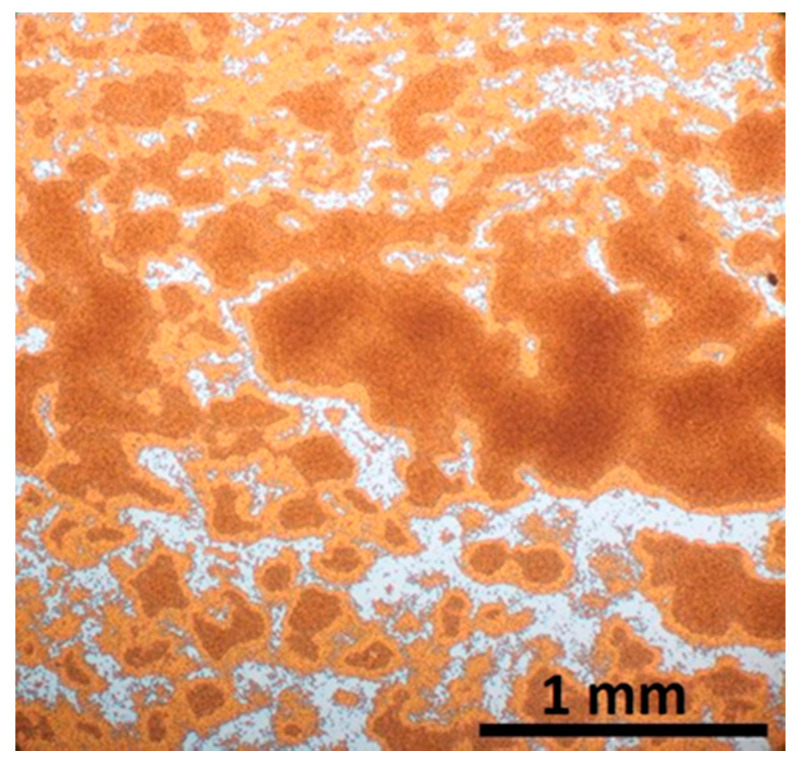
MSNs configurations obtained by drying in-situ application of a perpendicular magnetic field during spin-coating deposition of their aqueous solutions.

The in-situ process of the external field application perpendicular to the sample’s surface is followed while the SC routine is active. The magnetic field value is H = 0.1 T, and it is applied before Stage 4 of the SC routines. The resulting configuration is displayed in Figure 13. The analysis reveals that the MSNs, under the application of magnetic field, formed areas of multilayers, which appear denser (Figure 13). These, island-like, areas are separated by uncovered substrate areas. The dark brown areas, depicted in Figure 13, correspond to a greater number of piled-up layers, while the light brown areas correspond to monolayer. To note that a magnetized body placed in a uniform field will rotate to align its magnetic moment along field, but as the field then exerts two equal and opposite forces on its two “magnetic poles” there is no net force of translation. In an inhomogeneous field, on the other hand, it can be shown that a force proportional to the gradient of the field exists. Superparamagnetic particles, which do not possess a permanent magnetic moment but are magnetized by the existing field, experience a force proportional to the gradient of the square of the field. This mechanism can explain the tendency of the MSNs to accumulate as the already deposited magnetized spheres create a strong field gradient around them. Thus, the MSNs tend to concentrate on top of the existing islands forming multilayered structures while leaving uncovered substrate areas.

### 3.4. Spin Coating Followed by SDS Post-Treatment

**Figure 14:**
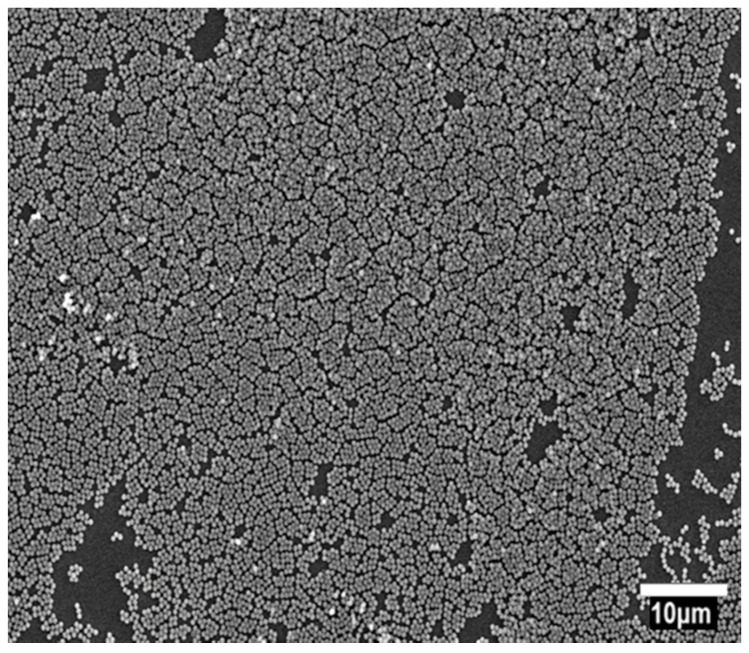
MSNs configuration obtained by spin-coating followed by dipping in sodium dodecyl sulfate (SDS) solution.

In this case, spin coating of the MSNs solution is followed by immersion of the covered substrates in a 10 wt.% SDS-aqueous solution at a small angle followed by lift-off from the SDS-aqueous surface. The samples are left to dry for 3 h in ambient conditions. This is a modification of the method by Rybczynski et al. using spin-coating instead of drop casting [25]. A two-stage spin coating has been employed, after the solution is dropped on the surface. The SC routine proceeds as follows: (a) the sample is rotated at 160 RPM/s for the solution to expand on the surface and (b) the rotation speed is ramped up to 800 RPM/s for 120 s, which was found as optimal. Large areas of hexagonal arrays are obtained with structural coherence spanning of 10–20 MSNs (Figure 14).

## 4. Conclusions

We have studied the self-assembly of paramagnetic polystyrene nanospheres arrays obtained by spin coating and drop casting of their aqueous solutions. It is shown that using superparamagnetic nanospheres, it is possible to control their self-organization by magnetic fields and achieve more complex, or even 3D structures, beyond the typical hexagonal arrays. Self-organization in the mesoscopic scale, where the thermal energy cannot help the system finding its energy minima, is an interesting problem where subtle play of several competing kinetic processes defines the outcome. The use of superparamagnetic spheres adds the long range magnetic dipolar interactions to the competing nanoparticle-surface (substrate hydrophobicity-hydrophilicity) and the nearest neighbor nanoparticle-nanoparticle interactions, which control order and agglomeration. An advantage of the superparamagnetic particles is that the strength of the interactions can be tuned by the applied field, and it is practically switched off when the external applied field is removed. The self-assembly of magnetically functional entities is much less studied compared to the vast existing literature of their non-magnetic counterparts. Magnetic forces favor agglomeration, which is usually not desirable in obtaining high-quality monolayers. However, it opens possibilities to obtain a richer variety of organized structures. The present work shows that, due to the long range of the magnetic forces, even weak fields can have a strong impact on self-organization patterns.

## Figures and Tables

**Table 1:** Spin coating routine summary.

Stage	RPM/s	Duration (s)	Phase	Effect
1	150	120		Solution spreads over substrate
2	250	120	Spin-up	Coverage improvement
3	800	60	Spin-off	Disordered monolayer formation
4	2500	20		MSNs adhesion to substrate
5	5000	20	Self-ordering	Hexagonal packing
6	8000	360	Drying	Enhanced hexagonal packing
